# Mechanisms of Hypoxic Up-Regulation of Versican Gene Expression in Macrophages

**DOI:** 10.1371/journal.pone.0125799

**Published:** 2015-06-09

**Authors:** Fattah Sotoodehnejadnematalahi, Karl J. Staples, Elvina Chrysanthou, Helen Pearson, Loems Ziegler-Heitbrock, Bernard Burke

**Affiliations:** 1 Department of Biology, School of Basic Science, Science and Research Branch, Islamic Azad University, Tehran, Iran; 2 Clinical & Experimental Sciences, University of Southampton Faculty of Medicine, Sir Henry Wellcome Laboratories, Southampton General Hospital, Southampton, United Kingdom; 3 Department of Infection, Immunity and Inflammation, University of Leicester, Leicester, United Kingdom; 4 EvA Study Center, Asklepios Fachkliniken and Helmholtz-Zentrum Muenchen, Gauting, Germany; 5 Dental School, College of Medical and Dental Sciences, University of Birmingham, Birmingham, United Kingdom; University of Dundee, UNITED KINGDOM

## Abstract

Hypoxia is a hallmark of many pathological tissues. Macrophages accumulate in hypoxic sites and up-regulate a range of hypoxia-inducible genes. The matrix proteoglycan versican has been identified as one such gene, but the mechanisms responsible for hypoxic induction are not fully characterised. Here we investigate the up-regulation of versican by hypoxia in primary human monocyte-derived macrophages (HMDM), and, intriguingly, show that versican mRNA is up-regulated much more highly (>600 fold) by long term hypoxia (5 days) than by 1 day of hypoxia (48 fold). We report that versican mRNA decay rates are not affected by hypoxia, demonstrating that hypoxic induction of versican mRNA is mediated by increased transcription. Deletion analysis of the promoter identified two regions required for high level promoter activity of luciferase reporter constructs in human macrophages. The hypoxia-inducible transcription factor HIF-1 has previously been implicated as a key potential regulator of versican expression in hypoxia, however our data suggest that HIF-1 up-regulation is unlikely to be principally responsible for the high levels of induction observed in HMDM. Treatment of HMDM with two distinct specific inhibitors of Phosphoinositide 3-kinase (PI3K), LY290042 and wortmannin, significantly reduced induction of versican mRNA by hypoxia and provides evidence of a role for PI3K in hypoxic up-regulation of versican expression.

## Introduction

Hypoxia (low oxygen tension) is a feature of many pathological tissues. The median oxygen tension in normal tissues is usually between 20 and 70 mmHg, but in ischemic pathological sites can be as low as zero mmHg [1]. Such hypoxic areas are found in tumours [2], wounds [3], atherosclerotic plaques [4], arthritic joints [5], and the retina [6] and ischemic limbs of diabetics [7].

Cells of the monocyte/macrophage lineage are involved in all of the above pathologies. Monocytes are derived from myeloid stem cells, and following release from the bone marrow circulate in the bloodstream for 1–3 days before migrating into tissues where they differentiate into macrophages [8, 9]. Macrophages are phagocytic, and can take up and destroy microorganisms or inhaled microscopic foreign bodies such as smoke, diesel exhaust, and pollen particles, and also have important roles in innate and adaptive immunity and tissue repair [10, 11]

It has been known for many years that macrophages accumulate in poorly vascularized, hypoxic sites [12]. Accumulation of macrophages has been reported in avascular, hypoxic and necrotic sites in breast [13] and ovarian carcinomas [14], wounds [15], atherosclerotic plaques [16] and arthritic joints [17].

Hypoxic macrophages up-regulate a number of hypoxia-inducible transcription factors, the most important of which is Hypoxia-inducible factor 1 (HIF-1) [18]. Macrophages are unusual in that they rely heavily on HIFs for energy generation and activity even under normal oxygen tensions [19], meaning that they are able to respond rapidly and effectively to the challenges posed by the need to function in hypoxic sites. Previous studies have shown that many genes are up-regulated in hypoxic macrophages [20, 21, 22, 23, 24]. The extra cellular matrix (ECM) proteoglycan versican has been identified as one such hypoxia-inducible gene [25].

Versican is a large aggregating chondroitin sulphate proteoglycan, and occurs in at least four isoforms [26]. It is found in various sites including the brain [27], and skin [28], and increased expression is observed in sites of tissue injury [29] and in cancers including breast [30], cervical [31], gastrointestinal tract, prostate [32], brain [33], and melanoma [34]. Several reports have also highlighted the role of versican in wound healing [35, 36] and in vascular disease, especially atherosclerosis [37, 38]. Versican binds low-density lipoprotein particles, and accumulation of versican in blood vessel walls is believed to promote extracellular lipoprotein retention and uptake leading to foam cell formation [39]. In the study which first reported hypoxic induction of versican [25], it was suggested to be regulated, at least in part, by Hypoxia-Inducible Factor 1 (HIF-1), the most important hypoxia-inducible transcription factor, which has been described as the “master regulator” of the transcriptional response to hypoxia.

The aims achieved in this study were to increase understanding of the mechanisms responsible for the up-regulation of versican by hypoxia in primary human macrophages, using promoter reporter deletion constructs, transcription factor over-expression, and gene expression quantification.

## Results

### Hypoxia induces versican gene expression in primary human monocyte-derived macrophages

**Fig 1:**
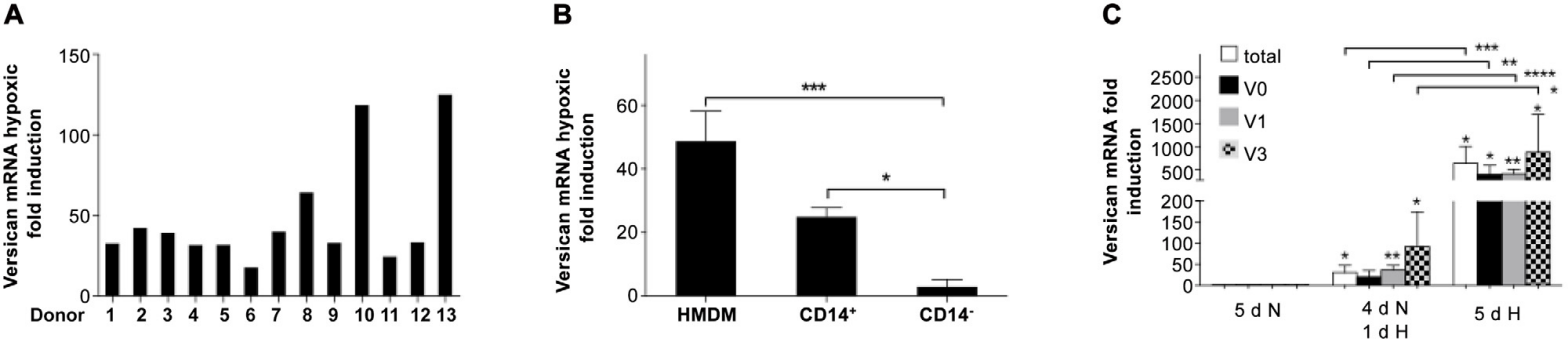
Up-regulation of versican gene expression by hypoxia in primary human macrophages. **(A)** Real Time RT-PCR quantification of the effect of 18hrs hypoxia (0.2% O_2_) on versican mRNA in 5-day differentiated HMDM from 13 different donors. Values are hypoxic fold induction relative to normoxia. **(B)** Changes in versican mRNA fold induction levels in response to 18hrs of hypoxia (0.2% O_2_) were quantified by real-time RT-PCR in HMDM, CD14^+^ magnetic bead purified monocyte-macrophages and CD14^-^ cells, all incubated for 5d after isolation before being exposed to a further 18h of either normoxia or hypoxia, in 3 independent experiments using different donors. Values are hypoxic fold induction relative to normoxia. **(C)** Real-time RT-PCR quantification of versican mRNA isoforms in HMDM after differentiation either 5d in normoxia (20.9% O_2_), 4d in normoxia followed by 1d in hypoxia, or 5d in hypoxia (0.2% O_2_), in 4 independent experiments using different donors. All data were normalized to 2MG mRNA levels determined by separate PCRs, and are expressed as mean fold induction (relative to the equivalent normoxic culture) ± SEM, and were analyzed for significance using paired t-tests. **** = p <0.0001, *** = p <0.001, * = p <0.05.

We investigated the effect of 18h hypoxia (0.2% O_2_ [1.5 mmHg]) on versican gene expression in 5-day differentiated primary human monocyte-derived macrophages (HMDM) using Real-Time RT-PCR. All 13 donors tested showed substantial hypoxic induction of total versican mRNA (using PCR primers which amplify all mRNA splice variants), however there was considerable variability (average 48 fold induction, range 20–120 fold; Fig 1A). The adherence method we used to isolate monocytes from blood yields a population of >95% monocyte-macrophages in our hands [18]. However, to confirm macrophages as the principle cell type showing hypoxic up-regulation of versican, we quantified versican induction in macrophages derived from monocytes isolated using MACS magnetic beads linked to antibodies specific for the monocyte surface antigen CD14. We compared these to adherence-purified HMDM and to the CD14-negative fraction of the MACS separation (found to consist of >95% lymphocytes as assessed by FACS analysis) from the same donors. All cells were incubated 5 days in normoxia before being exposed to a further 18h of either normoxia or hypoxia. Hypoxia significantly induced total versican mRNA expression in adherence-purified HMDM and CD14+ monocyte-derived macrophages but not in lymphocytes (Fig 1B).

While most studies on hypoxia are done with relatively short term exposure (typically 18-24h), we also wanted to model the behaviour of monocytes entering hypoxic tissue and undergoing differentiation into macrophages under hypoxia, since this is biologically relevant to pathological conditions in which chronic hypoxia is a feature and we have shown previously that this produces considerably higher fold induction of the VEGF gene than short term hypoxia [40]. We also wanted to study the effect of long and short term exposure to hypoxia on the mRNA levels of the different versican mRNA splice variants, which generate the distinct versican protein isoforms, to examine the possibility of differential expression. Monocytes were purified by adherence, and then cultured for 5 days under normoxia, or 4 days in normoxia followed by 1 day of hypoxia, or 5 days in hypoxia, as previously described [40]. One day of hypoxia produced a 30-fold induction of total versican mRNA, markedly less than 5d of hypoxia (637 fold) (Fig 1C). Analysis of the different splice variants of versican mRNA in HMDM showed that the V2 variant was not detectable, in line with previous findings in macrophages [41]. The three mRNA variants which were detectable (V0, V1, and V3) showed large average fold inductions: (20, 36 and 91-fold, respectively) after 1 day of hypoxia. However, after 5d of hypoxia, the fold inductions for all three variants were much higher (399, 407 and 878 fold respectively) (Fig 1C).

### Up-regulation of versican mRNA by hypoxia is not due to increased transcript stability

**Fig 2:**
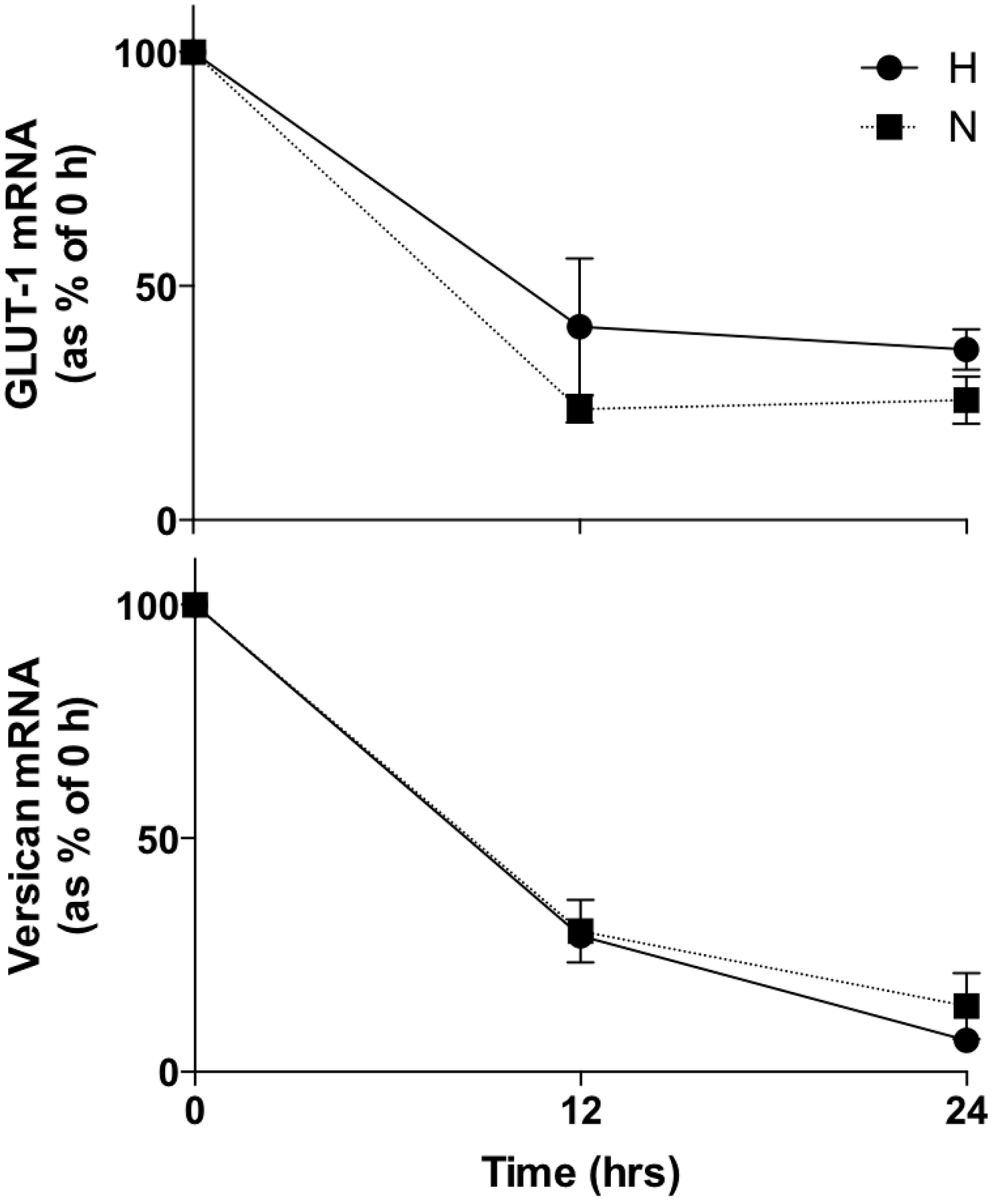
Assessment of versican mRNA decay in HMDM in normoxia and hypoxia by real-time RT-PCR after addition of Actinomycin D. N: normoxia 20.9% O_2_, H: hypoxia 0.2% O_2_. Data were normalized to 2MG mRNA levels. Data from 5 independent experiments are expressed as means ± SEM.

To determine whether up-regulation of versican mRNA by hypoxia is due to increased transcription or increased mRNA stability, we investigated the decay of versican mRNA and the mRNA of a gene known to be transcriptionally induced by hypoxia, Glucose Transporter 1 (GLUT-1), in normoxic and hypoxic primary HMDM (Fig 2). Messenger RNA degradation was not significantly different between normoxia and hypoxia for either gene, indicating that the increases in versican mRNA in hypoxic human primary macrophages (Fig 1A–1C) are due to transcriptional up-regulation rather than increased mRNA half-life.

### Hypoxic up-regulation of versican protein in human primary macrophages

**Fig 3:**
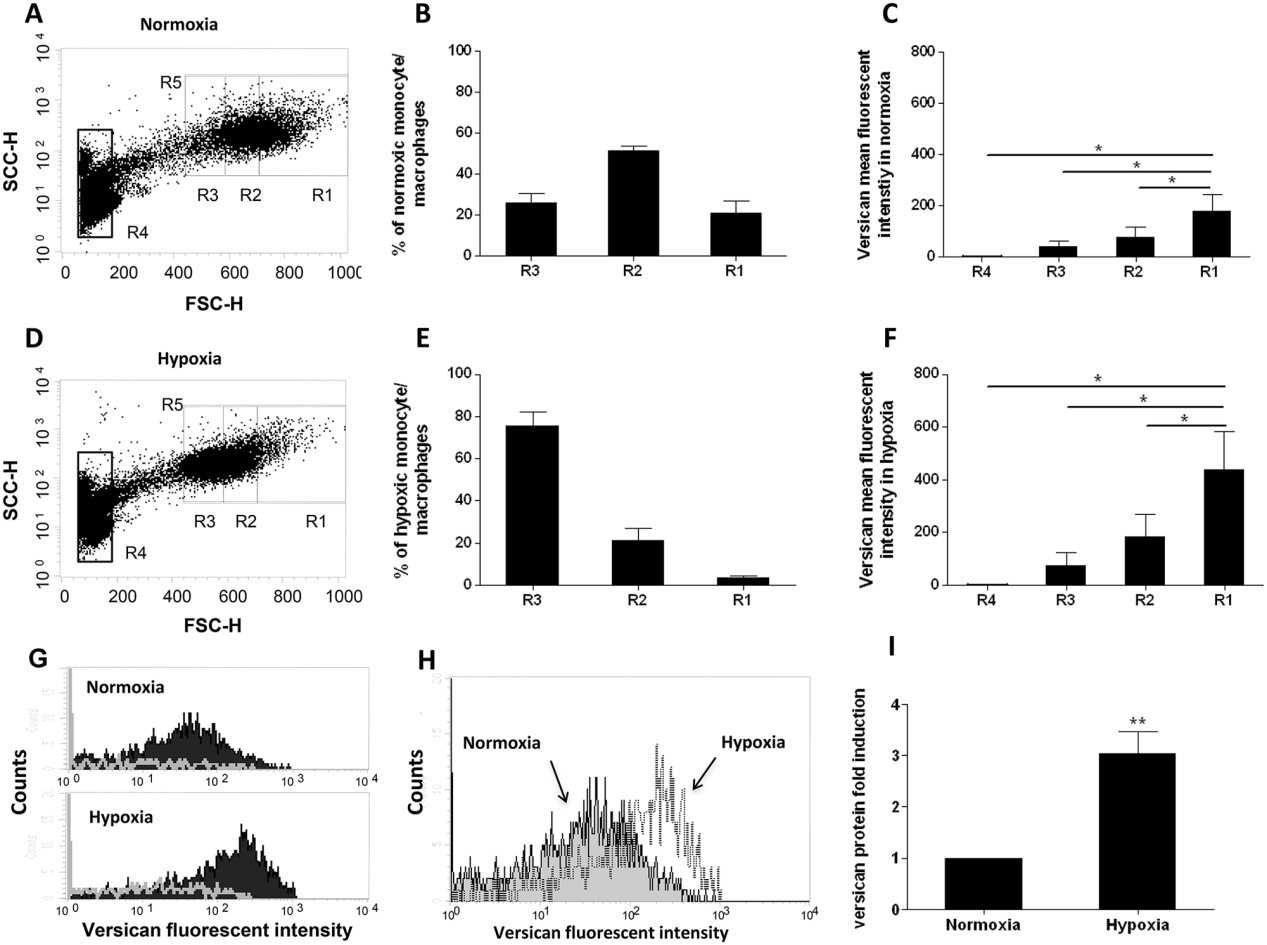
Quantitation of versican protein expression in monocytes / macrophages by flow cytometry. **(A and D)** Dot plot analysis of PBMC after 5 days in normoxia (20.9% O_2_; A) and hypoxia (0.2% O_2_; D). Monocyte/macrophages are subdivided into 3 regions R3-R1 in respect of increasing cell size (forward scatter). Lymphocytes are included in region 4. Region 5 encompasses all monocyte macrophages in Regions 1, 2, and 3. A representative example of 5 independent experiments is shown. **(B and E)** Percentage of the total monocyte/macrophage population (R5) present in regions R1, R2, and R3 in normoxia (B) and hypoxia (E). Data from 5 independent experiments are expressed as means ± SEM. **(C and F)** Versican mean fluorescent intensity in regions 1, 2, 3 and 4 in Normoxia (C) and Hypoxia (F). Data from 5 independent experiments are expressed as means ± SEM. **(G)** Histogram of the fluorescent intensity with a versican specific antibody (black fill) compared to the isotype control antibody (white line) in region R1 cells in Normoxia and Hypoxia. A representative example of 5 independent experiments is shown. **(H)** Histogram analysis of the versican fluorescent intensity in region R1 cells in Normoxia (shaded) and Hypoxia (clear). A representative example of 5 independent experiments is shown. **(I)** Versican protein fold induction in cells region R1 in normoxia and hypoxia. Data from 5 independent experiments are expressed as means ± SEM. For panels B, E, C, F, and I, the normoxic value in each experiment was assigned an arbitrary value of 1. Data were further analyzed using two-tailed, paired t-tests. ** p < 0.01,* = p <0.05.

To investigate whether the hypoxia-induced increases in versican mRNA were matched by increases in versican protein, we analyzed versican protein expression in PBMC—derived macrophages after 5 days incubation in normoxia or hypoxia using intracellular staining followed by flow cytometry [42, 43, 44]. Since versican is exported, all cells were treated with Brefeldin A to block protein export in order to facilitate quantification by intracellular staining. With this approach we can demonstrate that hypoxia induces a clear increase in intracellular versican protein (Fig 3).

When looking at macrophages with different forward scatter signals we noted that cells with high forward scatter (large macrophages) gave a higher specific mean intensity compared to cells with a low forward scatter (small macrophages). To analyse this, the macrophage populations were divided into 3 separate regions (R3-R1) on the basis of increasing forward scatter (FSC), as shown in Fig 3A and 3D. The proportion of cells in each of these regions differed in normoxia and hypoxia (Fig 3B and 3E), likely reflecting the inhibitory effect of prolonged hypoxia on the increase in cell size which is associated with macrophage maturation [40]. In both normoxia and hypoxia, increasing cell size (forward scatter) significantly correlated with increasing versican expression (Fig 3C and 3F). Comparison of R1 for normoxic and hypoxic macrophages (Fig 3G) demonstrates a statistically significant overall increase in versican protein expression of approximately 3 fold (Fig 3H). Similar hypoxia-induced increases in versican mean fluorescent intensity were also observed in the smaller macrophages in regions R2 and R3 (Fig 3F).

Very low levels of versican protein expression, and no hypoxic induction, were detected in the lymphocyte population (R4; Fig 3C and 3F), which correlates well with the low versican mRNA level, and relatively low and non-significant hypoxic induction observed in lymphocytes (Fig 1B).

### Deletion analysis of the versican promoter

**Fig 4:**
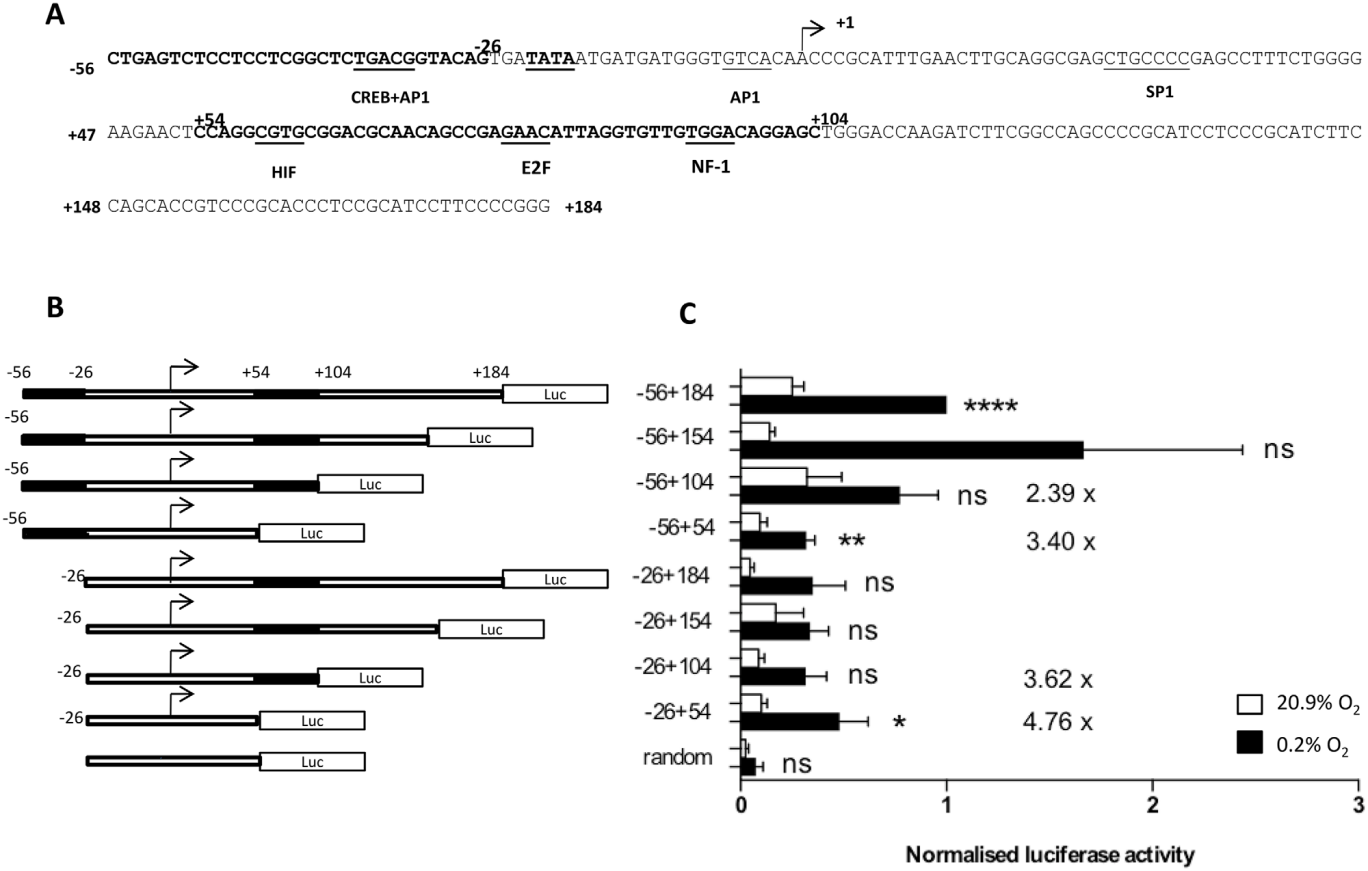
Activity of versican promoter luciferase reporter constructs in normoxia and hypoxia in HMDM. **(A)** Putative transcription binding sites for hypoxia inducible factor (HIF), cAMP responsive element binding (CREB), activator Protein 1 (AP1), SP1, nuclear factor 1 (NF-1) and E2F within the 240 bp (-56+184) versican promoter sequence. The -56 to -26 and +54 to +104 sequences which are important for high level expression are in bold. **(B)** Schematic diagram of versican promoter or random 26mer luciferase reporter constructs used. **(C)** activity of versican promoter or random 26mer luciferase reporter constructs in HMDM after 5d incubation in normoxia (20.9% O_2_), or hypoxia (0.2% O_2_). Data from an average of 6 independent experiments with each construct, minimum n = 3 for each construct, are expressed as means ± SEM. Luciferase data were normalized to protein levels. Data assessed for significant increase in hypoxia compared to random construct control using two-tailed t tests, *** = p <0.001 ** p < 0.01,* = p <0.05, ns = not significant.

To investigate the possible mechanisms of hypoxic up-regulation of the versican promoter, we carried out a search of the proximal promoter region (-56 to +184; Fig 4A) for putative binding sites for hypoxia-inducible transcription factors, using Genomatix Matlnspector and TESS software. The -56+184 region of the versican promoter was chosen for analysis because it has previously been shown to constitute a functional promoter and contains a typical TATA box and has putative binding sites for a number of transcription factors including CREB, AP-1 and SP1 [35, 45]. An analysis of this sequence using Matinspector software identified a putative binding site at +60 for the transcription factor Hypoxia-inducible factor-1 (HIF-1) (Fig 4A). This putative HIF-1 site or Hypoxia Response Element (HRE) [46] was not identified in the initial report of versican hypoxia responsiveness [25]. To analyse whether parts of this proximal promoter region are required for high level activity of the versican promoter in hypoxia, we generated a series of deletion constructs in a luciferase reporter plasmid (Fig 4B) and transfected them into HMDM. Promoter constructs containing the sequences -56+184, -56+154 and -56+104 (all containing the putative HIF-1 site at +60) show high levels of expression in both normoxia and hypoxia (Fig 4C). Luciferase activity dropped markedly in the -56+54 construct, indicating that the region +54 to +104, which includes the putative HIF binding site at +60bp, is important for high level expression of these versican promoter constructs in both high and low oxygen concentrations. However, hypoxic induction (5.7 fold) was still observed with the -56+54 construct, despite its lack of a HIF site. Fig 4C also shows that luciferase expression is markedly lower in constructs beginning at -26 (regardless of whether they contain the putative +60 HIF site or not) compared to those beginning at -56, indicating that the region from -56 to -26 encompasses a separate region required for high level activity of these versican promoter reporter constructs.

### Role of HIF-1 in hypoxic induction of versican expression in HMDM

**Fig 5:**
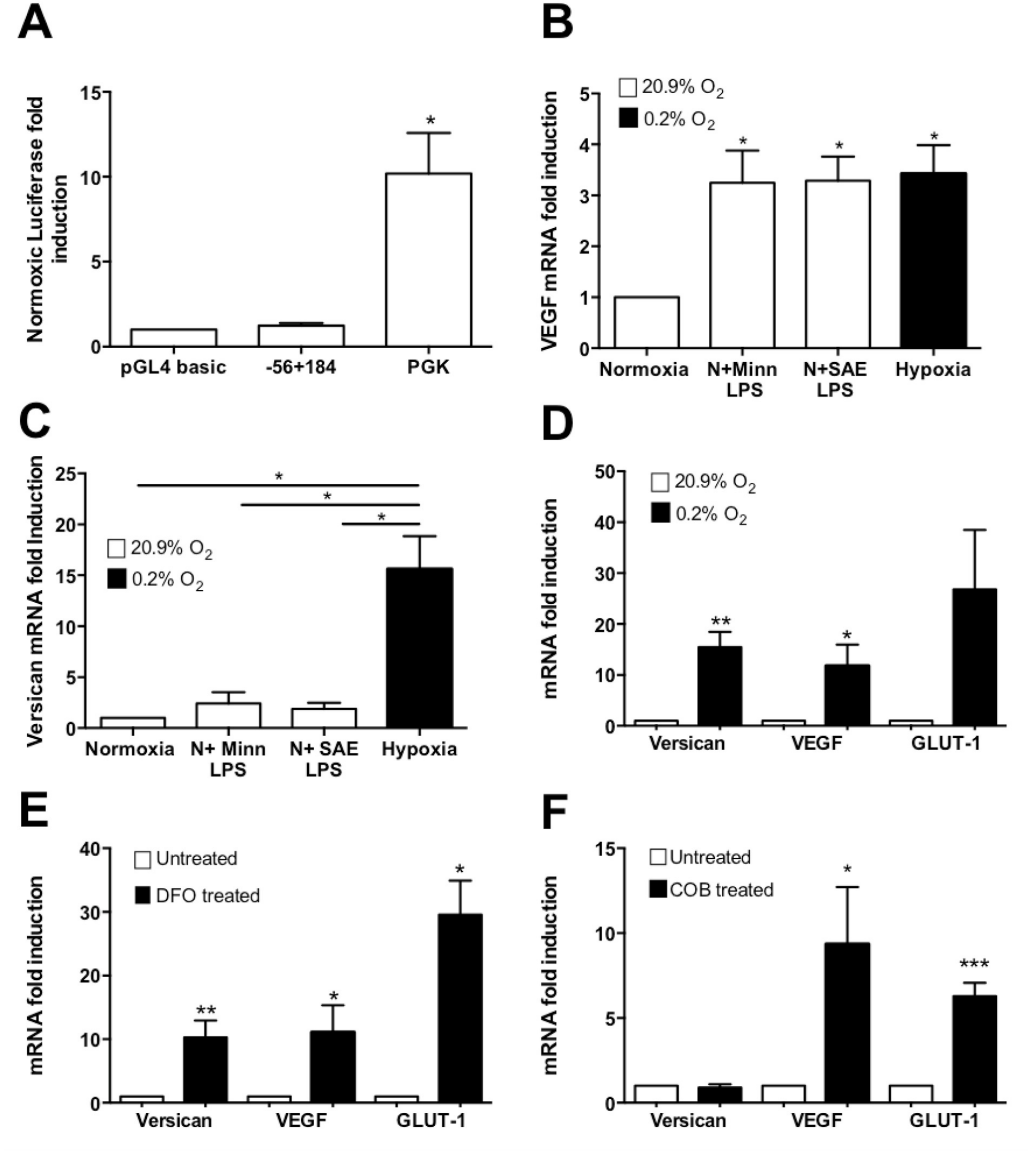
Investigation of the role of Hypoxia Inducible Factor 1 (HIF-1) in versican up-regulation. **(A)** Effect of over-expression of HIF-1α on the 240 bp (-56+184) versican promoter construct in HMDM. PGK was used as a positive control and pGL4 basic as a negative control. **(B and C)** Real time-PCR analyses showing VEGF and versican mRNA fold induction after treatment with two different preparations of LPS (MINN LPS, Salmonella Minnesota LPS; SAE LPS, Salmonella abortus equii LPS) in comparison with hypoxia. **(D)** Real time-PCR analyses show versican, VEGF, and GLUT-1 mRNA fold induction in hypoxic and normoxic HMDM. **(E)** Real time-PCR analyses of versican, VEGF, and GLUT-1 mRNA fold induction in HMDM treated with cobalt chloride (COB). N: normoxia 20.9% O_2_, H: hypoxia 0.2% O_2_. All incubations, with or without hypoxia, were for 18hrs. Data were normalized to 2MG mRNA levels. Data from 3 (A, B, and C) or 5 (D and E) or 8 (F) independent experiments are expressed as means ± SEM. Data were further analyzed using paired two-tailed t-tests. *** = p <0.001 ** p < 0.01,* = p <0.05

To investigate whether the putative HIF binding site at +60 is responsive to HIF-1, we over-expressed HIF-1α in normoxic HMDM transfected with the -56+184 versican promoter luciferase reporter construct. As Fig 5A shows, HIF-1α over-expression did not affect the expression of this construct, in contrast to marked up-regulation of the control PGK-1 reporter construct which is known to be regulated by HIF-1 [47, 48, 49].

We also investigated the role of HIF-1 in endogenous versican mRNA expression. Previous studies have shown that treatment of normoxic macrophages with LPS induces HIF-1α mRNA and protein, and consequently HIF-1 inducible genes including Vascular Endothelial Growth Factor (VEGF) [50, 51]. VEGF up-regulation by LPS has been shown to be dependent on HIF-1 up-regulation [52, 53]. We treated normoxic HMDM with LPS and quantified VEGF and versican mRNA levels. As expected, LPS induced the mRNA of the HIF-1-up-regulated gene VEGF (Fig 5B), however it did not induce versican mRNA in the same samples (Fig 5C), suggesting that HIF-1 up-regulation alone (in the absence of other factors such as hypoxia itself) is not sufficient for significant induction of versican.

To further elucidate the role of HIF-1 in the hypoxic induction of versican gene expression, HMDM were treated with the “hypoxia mimetic” agents Desferrioxamine (DFO) and CoCl_2_. DFO and CoCl_2_, which are chemically unrelated, act via different mechanisms to stabilise the hypoxia-inducible α subunit of HIF-1 in normoxia, leading to induction of HIF-1 dependent hypoxia-inducible genes [54, 55, 56]. Among the genes they induce are VEGF and GLUT-1 [57, 58, 59]. Determination of versican, VEGF and GLUT-1 mRNA levels by real time RT-PCR showed that as expected, 18h hypoxia induced versican (15.5-fold), VEGF (11.7-fold) and GLUT-1 mRNA (15.5-fold) (Fig 5D). Similarly, DFO also induced versican mRNA expression in normoxia (10-fold), and VEGF (11-fold) and GLUT-1 (29.5-fold), in the same RNA samples (Fig 5E). In contrast, CoCl_2_ markedly induced VEGF (10.6-fold) and GLUT-1 mRNAs (6.3-fold) but not versican mRNA, in the same RNA samples (Fig 5F).

**Fig 6:**
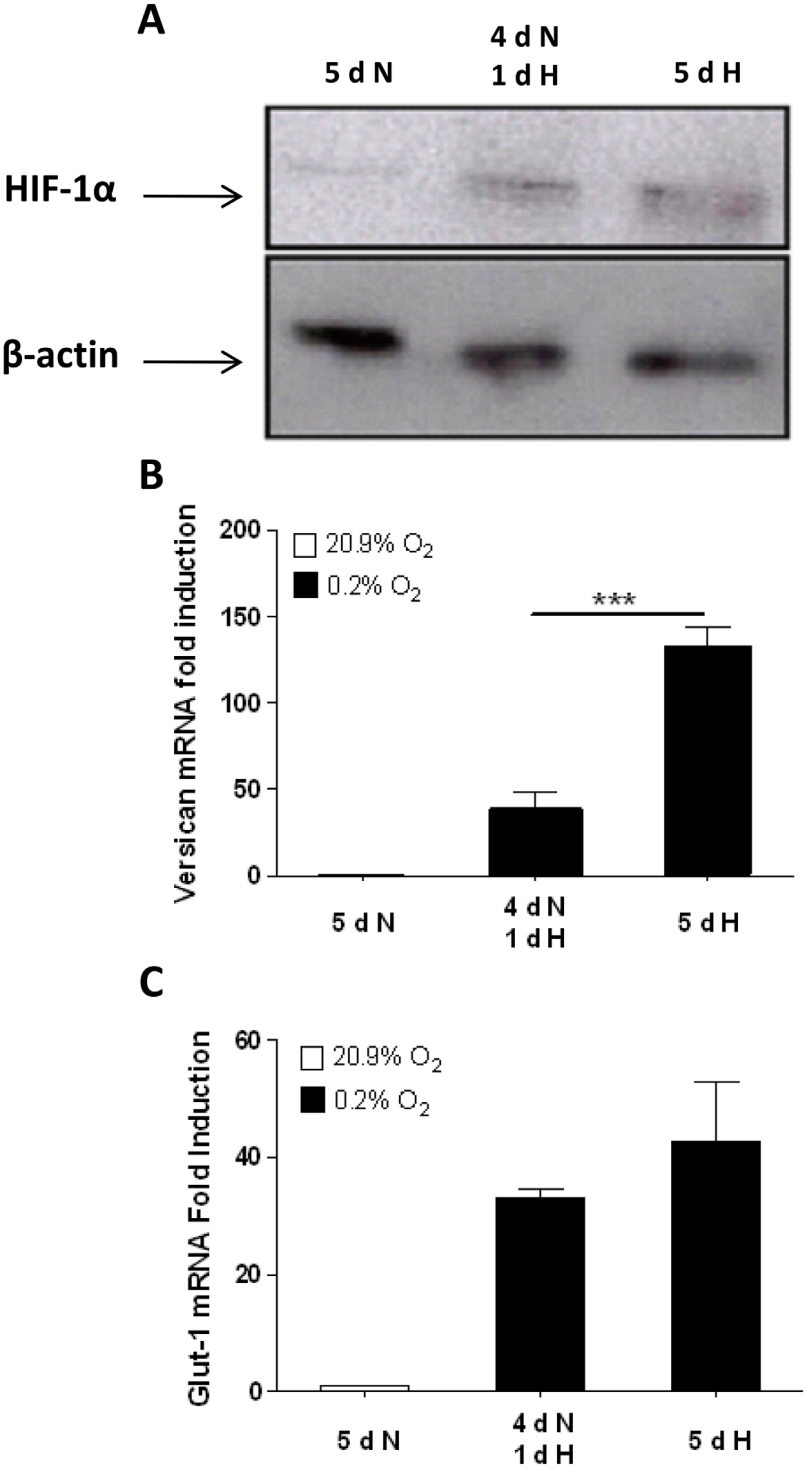
Immunoblotting shows lack of correlation between HIF-1α protein level and versican mRNA up-regulation by hypoxia. **(A)** After incubation under the conditions indicated, cell lysates were prepared from HMDM and immunoblotted for HIF-1α and actin. A blot representative of 3 independent experiments is shown. **(B and C)** Real time-PCR analyses show versican and GLUT-1 mRNA fold induction after 5 days of normoxia, 4 days of normoxia followed by 1 day of hypoxia, or 5 days of hypoxia. N: normoxia (20.9% O_2_), H: hypoxia (0.2% O_2_). Data were normalized to 2MG mRNA levels. Data from 3 independent experiments are expressed as means ± SEM. The normoxic value in each experiment was assigned an arbitrary value of 1. Data were further analyzed using two-tailed, paired t-tests. *** p< 0.001

Since cobalt chloride is known to induce HIF-1 protein and consequently expression of HIF-1 controlled genes [60], the data indicate that versican is regulated differently to VEGF and GLUT-1, via mechanisms which can be activated by hypoxia and DFO but not by CoCl_2_, again suggesting that HIF-1 is not sufficient in itself to induce the versican promoter. Therefore, we compared the level of HIF-1α protein (Fig 6A) with versican (Fig 6B) and the classical HIF-1 regulated gene GLUT-1 (Fig 6C) mRNA levels after 5 days in normoxia, or after 4 days in normoxia followed by 1 day of hypoxia, or after 5 days of continuous hypoxia. Versican mRNA levels did not correlate closely with the levels of HIF-1α protein, being significantly higher after 5 days, in contrast to the mRNA level of GLUT-1. These results cannot be explained by versican mRNA having greater stability in hypoxia than GLUT-1 mRNA, as Fig 2 showed that the reverse is true: GLUT-1 mRNA is marginally more stable than versican mRNA in both normoxia and hypoxia. The data therefore suggest that versican hypoxic mRNA levels may be responsive to factors other than HIF-1.

### Role of the PI3k—β-catenin pathway in hypoxic induction of versican expression in HMDM

**Fig 7:**
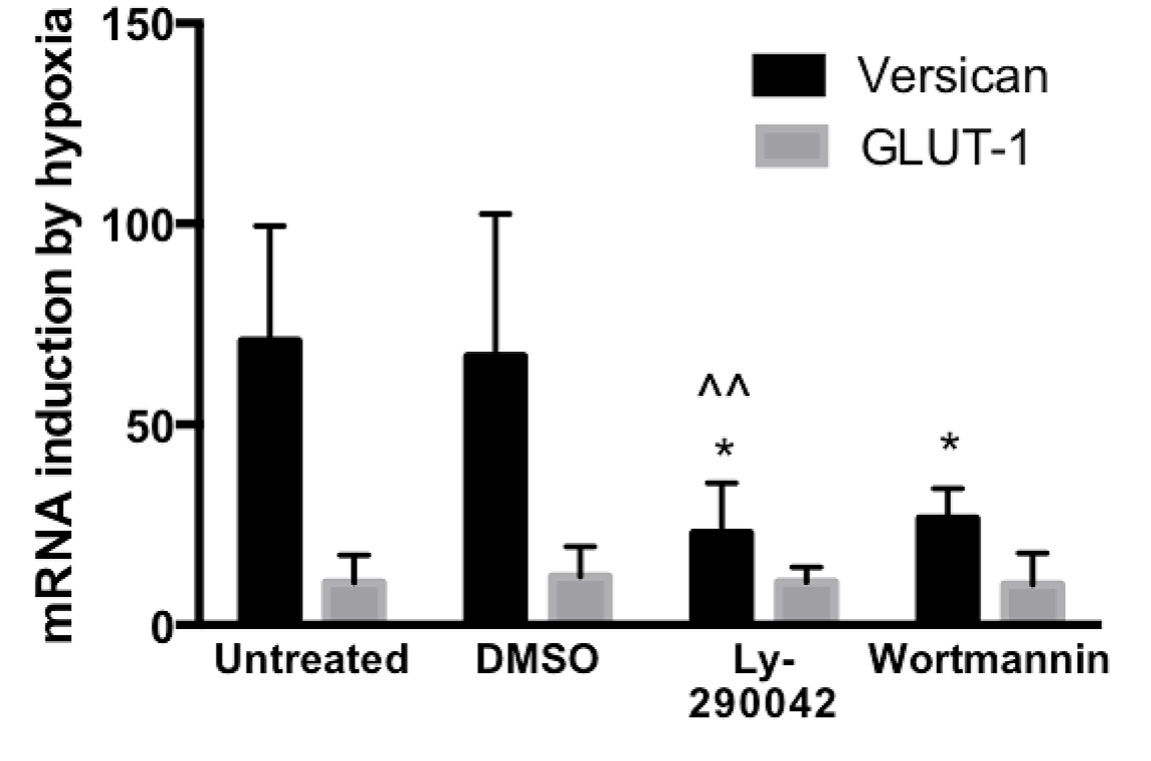
Real Time RT-PCR analysis of the effect of PI3K inhibitors on induction of versican and GLUT-1 mRNAs by 18h of exposure to hypoxia (0.2% O_2_). LY290042 was used at 2μM and wortmannin at 300μM. ^^; p<0.05 compared to DMSO control, *; p<0.05 compared to untreated control, ratio paired t test, one tailed. Data from 5 independent experiments using HMDM from different donors, expressed as means ± SEM.

Data from the above experiments using CoCl_2_ and DFO suggested involvement of a signalling pathway which can be activated by hypoxia and DFO but not by CoCl_2_. One such pathway is known, involving PI3-kinase regulation of the transcription factor β-catenin. Hypoxia [61] and DFO [62, 63] have been demonstrated to up-regulate this PI3K—β-catenin pathway, whereas cobalt chloride down-regulates it [64]. Moreover, versican has previously been shown to be up-regulated by the PI3K—β-catenin pathway [65]. To investigate the possible role of the PI3K pathway in hypoxic up-regulation of versican, HMDM were treated with two distinct specific inhibitors of PI3K, LY290042 and wortmannin. These treatments resulted in significantly reduced hypoxic inductions (Fig 7). In contrast, PI3-Kinase inhibitors did not affect induction of GLUT-1, a classical HIF-1 regulated gene. These data suggest a possible role for PI3-Kinase in hypoxic up-regulation of versican mRNA in HMDM.

## Discussion

Versican, an abundant and widely expressed extracellular matrix proteoglycan, has important roles in a number of pathological conditions including cancer and atherosclerosis [30, 31,32, 33,34, 37, 38], in which accumulation of macrophages in hypoxic sites is a feature. In the present study we aimed to characterize the expression of versican by macrophages and examine the mechanisms responsible for its hypoxic induction.

Interestingly, our data show that in human monocyte-derived macrophages (HMDM), extended periods of hypoxia (5 days) produce far higher versican mRNA fold inductions (over 500 fold on average) than have been previously reported following 1 day of hypoxia (40 fold [20]). This is of crucial biological relevance because macrophages entering hypoxic tissues such as tumours, wounds and atherosclerotic plaques, where they accumulate in very high numbers, remain there for extended periods [58, 16]. Thus, the degree of up-regulation of versican in such sites, and the role played by hypoxia in versican upregulation in pathological sites, is likely to be considerably more significant than previously appreciated. Our data also show that these large increases in expression are observed for all detectable versican splice variants (V0, V1 and V3; Fig 1C).

Actinomycin D mRNA decay experiments demonstrated that hypoxic induction of versican mRNA in macrophages does not occur via changes in mRNA stability in hypoxia, since mRNA decay did not differ between 0.2% and 20.9% O_2_, indicating that versican mRNA induction by hypoxia occurs by transcriptional up-regulation (Fig 2).

We also investigated hypoxic up-regulation of versican at the protein level. Intracellular FACS analysis of PBMC showed induction of versican protein in human monocyte/macrophages after 5 days in hypoxia, albeit at a much lower fold induction than for the mRNA, and showed that versican protein production is specific to monocyte-macrophage lineage cells and is not detectable in lymphocytes (Fig 3).

Asplund et al. [25] presented evidence which they believed implicated the hypoxia-inducible transcription factor HIF-1 as being involved, at least in part, in hypoxic up-regulation of versican. Co-localisation of versican and HIF-1α was reported in immunostained serial sections of human carotid lesions. However, this is data does not prove a direct link: many other hypoxia-inducible transcription factors and other hypoxia-inducible proteins also co-localise in hypoxic sites, regardless of their mechanism of up-regulation. Therefore, we decided to investigate the possible role of HIFs in versican hypoxic up-regulation in more detail. Asplund et al. reported a conserved putative HIF-1 binding site in the +2871 to +2888 region of the versican gene in 7 species. We performed a more wide-ranging search of 37 species in the ENSEMBL database, which shows that although some species do indeed possess a putative HIF-binding Hypoxia Response Element (HRE) in or near this position, a majority (22/37) either have a very weak consensus HIF site (2/37) or no HIF site (20/37). The finding that the majority of species which could be analysed do not contain a HRE at this site weakens the evidence for this being a functionally conserved transcription factor binding site, and suggests that it may be simply a chance occurrence or a contributory rather than an essential site of predominant importance. Anti-HIF-1α siRNA experiments carried out by Asplund et al using the THP-1 monocytic leukemia cell line showed only a minor reduction in versican mRNA in hypoxia (approximately 20%) compared to approximately 40% reduction for the known HIF-1 regulated gene Glut-1. This data led Asplund et al [25] to suggest that versican may be only partly regulated by HIF-1. Their HIF-2α siRNA experiments produced a 27% knock-down in hypoxic versican mRNA levels, and combined HIF-1α and HIF-2α siRNA showed only 44% reduction in versican mRNA. Overall, given that macrophages have long been known to rely heavily on HIFs for energy production under both normoxia and hypoxia because of their dependence on glycolysis [19], many mRNAs are likely to be down-regulated non-specifically and indirectly by siRNA knockdown of HIFs. The specificity of the modest reductions in hypoxic versican mRNA levels observed in the Asplund et al. siRNA experiments cannot be confirmed without suitable controls, such as the demonstration that a gene which is known to be HIF-independent is unaffected by siRNA treatment. Since no such controls were shown [25], these data are not conclusive.

To investigate the regulation of versican transcription in HMDM, we cloned the -56 to +184 versican promoter sequence previously identified as constituting a functional promoter [35, 45] into the luciferase reporter plasmid pGL4. Analysis of this promoter sequence revealed binding sites for a number of transcription factors which are known to be hypoxia-inducible: CREB and AP-1 at -34, AP-1 at -4, SP1 at +25, HIF-1 at +60, E2F at +80 and NF1 at +94 (Fig 4A). We made a series of promoter deletion reporter constructs and transfected them into HMDM. In general, promoter activity was higher in hypoxia than in normoxia (Fig 4C), however overall fold induction (H/N ratio) did not vary greatly between mutants. Promoter activity was lower in the -56 to +54 construct compared to constructs containing the region +54 to +104, indicating that the +54 to +104 sequence is crucial for high level versican promoter activity in both normoxia and hypoxia (Fig 4B). The data also showed that promoter activity is markedly reduced in constructs which lack -56 to -26, suggesting that this region also contains binding sites for transcription factors which are important for versican promoter activity in HMDM. Overall, the data indicate that both the -56 to -26 and +54 to +104 regions are required together in the same construct for high expression from these versican promoter constructs in HMDM.

These observations suggest two alternative mechanisms: either two or more independent transcription factors bind at these two sites, and are necessary for high level transcription, or factors binding in these two regions interact to facilitate transcription; candidates include AP-1 (-34 and -4) which can interact with NF-1 (+94) [59] or CREB (-34) which could interact [66] with the putative binding site we have identified at +60 for HIF-1, a transcription factor which has previously been suggested [25] to play a role in versican hypoxic induction.

To examine the functionality of this putative HIF-1 binding site, we cotransfected the -56+184 versican promoter construct with a HIF-1α over-expression construct; however, no induction was observed, in contrast to the HIF-1 responsive control PGK-1 construct which was strongly induced (Fig 5A).

To further elucidate potential role of HIF-1 in hypoxic up-regulation of versican, we examined induction of versican mRNA in HMDM. Previous studies [50, 51] have shown that HIF-1α mRNA and protein are up-regulated by lipopolysaccharide (LPS) in monocyte-macrophage lineage cells. Our data show that treatment of HMDM with LPS from two different bacterial species did not significantly induce versican mRNA, in contrast with the mRNA of VEGF, which is known to be up-regulated by LPS via a HIF-1-dependent mechanism [50] (Fig 5B and 5C). In addition, we treated HMDM with cobalt chloride and Desferrioxamine (DFO). These “hypoxia mimetic” agents act via different mechanisms to stabilise HIF-1α, increasing HIF-1 protein levels in normoxia, and up-regulating the expression of HIF-1 dependent hypoxia-inducible genes [54, 55, 56, 60]. Both hypoxia and DFO up-regulated versican and the two control HIF-1 dependent genes VEGF and GLUT-1. In contrast cobalt chloride up-regulated VEGF and GLUT-1 but not versican mRNA (Fig 5D–5F). Taken together, the lack of induction of versican mRNA by LPS and cobalt chloride, two stimuli which are known to strongly up-regulate HIF-1α and thus HIF-1-regulated gene expression, suggest that versican is up-regulated differently to VEGF and GLUT-1, either via a mechanism in which HIF-1 alone is not sufficient or via a HIF-1 independent mechanism.

The ability of DFO, but not cobalt chloride, to up-regulate versican mRNA suggested the possibility of a hypoxic induction pathway involving up-regulation of β-catenin by PI3K, because this pathway is known to regulate versican expression [65], and to be induced by hypoxia and DFO [61, 62] but not induced by cobalt chloride [64]. To test this hypothesis we treated HMDM with two distinct PI3K inhibitors, LY290042 and wortmannin, and showed that both caused a marked reduction in hypoxic induction of versican while having no effect on hypoxic induction of the classic HIF-1 regulated gene GLUT-1 (Fig 7). These data provide evidence of a role for PI3K in the hypoxic induction of versican in HMDM. Based on the previous work of Rahmani et al., [65], this could be mediated via the stimulatory effects of PI3K on β-catenin. This concurs with the evidence of our promoter deletion studies, in which even a very minimal reporter construct (-26 to +54) was inducible by hypoxia (Fig 4), since there is an AP-1 binding site at position -4, and β-catenin has been shown to be able to mediate transcriptional up-regulation through AP-1 sites, in combination with TCF/LEF and ATF transcription factors [67]. In addition to the previously determined ability of β-catenin to regulate the versican promoter by modulating TCF/LEF transcription factor activity [61], a recent study by Yang and Yee et al., [68] has reported a novel versican- β-catenin control mechanism involving shared regulatory miRNAs. Precise delineation of the potential role of PI3K in hypoxic induction of versican, which has been highlighted in this study, and its relative importance, merits further investigation.

## Materials and Methods

### Ethics Statement

Blood for preparation of peripheral blood mononuclear cells was obtained from healthy volunteer donors. Informed written consent was obtained from all donors and the study was approved by the local research ethics committee of the Department of Infection, Immunity and Inflammation, University of Leicester, Leicester, UK.

### Isolation and hypoxic culture of peripheral blood mononuclear cells (PBMC) and human monocyte derived macrophages (HMDM)

PBMC were isolated from heparinised (10 U/ml) blood by centrifugation on Ficoll-Paque Plus (Amersham Biosciences, Little Chalfont, UK) according to the manufacturer’s instructions. Cells were resuspended at a density of 1 × 10^6^ cells/ml in Iscove’s-modified Dulbecco’s medium (Sigma, Poole, UK) supplemented with 2.5% human AB serum (BioSera, UK), 2 mM L-glutamine (Sigma, UK), 200 U/ml penicillin and 200 μg/ml streptomycin (Sigma, UK). For Real Time PCR experiments, PBMC were cultured in 2ml at a cell density of 2×106 per well in 6 well plates (Nunclon) and for FACS experiments they were cultured in 6 well Costar ultra low attachment plates (Corning, USA). Adherence-purified Human Monocyte Macrophages (HMDM) in 6 well plates (Nunc) were obtained as previously described [21] by allowing PBMC to adhere to wells for 2 hours at 37°C. Non-adherent cells were removed by medium changing. The adherent monocytes were then incubated for 5 days in 37°C to allow differentiation into macrophages. For transfection experiments, PBMC were cultured in a volume of 4 ml at a density of 1×106 cells per ml in 6 well in ultra low attachment plates and then cultured for 5 days to allow differentiation of monocytes into macrophages. CD14-microbead-purified monocytes were prepared using CD14 microbeads and MS isolation columns from Miltenyi Biotec (Bisley, UK), following the manufacturer’s instructions, and cultured in 6 well adherent plates at a cell density of 1x10^6^ cells /ml, in 2 ml /well. Cells which passed through the column were designated CD14- cells and were found to be typically >95% lymphocytes as assessed by FACS analysis. Cells were cultured under normal oxygen concentrations (normoxia) in a humidified atmosphere of 95% air (20.9% O_2_), 5% CO_2_, which, allowing for partial pressure of water vapour, equates to 18.6% O_2_ (141 mmHg), or under hypoxia in a humidified multi-gas oxygen control Galaxy R incubator (New Brunswick Scientific, UK) containing 5% CO_2_, 94.8% N_2_, and 0.2% O_2_ (1.5 mmHg). Oxygen levels indicated on the incubator display screens were verified using a separate oxygen meter (Analox, UK).

### Actinomycin D treatment

For mRNA half-life analysis, in order to block further transcription, Actinomycin D (Sigma) at a final concentration of 10μg/ml was added to 2 × 10^6^ adherent HMDM after incubation for 5 days in either normoxia or hypoxia. Cells were then incubated under normoxia or hypoxia for the required times prior to RNA isolation.

### LPS, Cobalt chloride and Desferrioxamine treatment

After 5 days incubation under normal oxygen tensions, 2 × 10^6^ adherent HMDM were treated with *Salmonella abortus equii* lipopolysaccharide (Alexis Biochemicals, Lausen, Switzerland) or *Salmonella minnesota* lipopolysaccharide (Sigma) at 100 ng/ml and incubated under normoxia or hypoxia for the required time prior to RNA isolation. Cobalt chloride (Sigma) was added to cell culture medium at a final concentration of 300 μM, and Desferrioxamine (DFO; Sigma) was added at a final concentration of 200 μM.

### PI3K inhibition

Cells were treated with LY290042 (Sigma) at final concentration of 2μM and wortmannin (Sigma) at a final concentration of 200nM. Inhibitors were dissolved in DMSO (Sigma).

### RNA isolation and real-time RT-PCR

RNA was isolated using TRI reagent (Sigma) according to the manufacturer’s instructions. Reverse transcription was carried out as previously described [62]. Reverse transcription products were amplified in a 20 μl reaction mix containing 1 × SYBR Green Taq ReadyMix Capillary Formulation (Sigma) and the required forward and reverse primers.

Versican primer sequences were derived from NCBI nucleotide bank accession no. NM_004385. Forward primer 5′-ACAAGCATCCTGTCTCACG-3′ is from nucleotides 9879–9897; reverse primer 5′-TGAAACCATCTTTGCAGTGG-3′ is from nucleotides 10254–10273. β2MG[18]: Fwd5′-GGCTATCCAGCGTACTCCAAAG-3′; Rev 5′-CAACTTCAATGTCGGATGGATG-3′. GLUT-1 and VEGF primers are as previously described [21]. Amplifications were carried out on a Roche LightCycler Real-time PCR instrument (Mannheim, Germany) using the following cycling parameters: pre-incubation at 95°C for 10 min then 45 cycles of 96°C for 10 sec, 60°C for 10 sec, 72°C for 25 sec. All reactions were finished with a melting curve run to establish specificity. PCR data were normalized to the relative concentration of β_2_-microglobulin (β2MG) housekeeping gene mRNA determined by separate PCR on each sample [40]. In contrast to versican mRNA levels, which were highly up-regulated by hypoxia, β2MG mRNA levels in HMDM were not markedly affected by the hypoxic conditions used (0.2% O_2_ for up to 5 days), as we also reported in a previous study [40], in which we also found that β2MG levels correlated closely with total RNA quantification values.

### Generation of versican promoter reporter constructs

A 240-bp (-56/+184) versican promoter sequence and shorter versions thereof were generated by PCR from human genomic DNA with the appropriate sets of primers based on the published versican promoter sequence ([45], accession number U15963; primer sequences available upon request). These inserts were cloned into the SfiI site of the pGL4.10 [luc2] luciferase reporter plasmid (Promega) using standard techniques. A 29 bp random nucleotide sequence (CTAGCTAGCTAGCTAGCTAGCTAGCTAGC) was generated and also cloned into pGL4.10 [luc2] for use as a negative control in transfection experiments. All constructs were verified by sequencing.

### Transfection and Luciferase Activity Assays

PBMCs were transiently transfected with μg of reporter plasmid DNA using JetPEI transfection reagent (Polyplus) according to the manufacturer’s protocol. Transfected cells were plated at 2×106 cells per well in 6 well plates (Nunc). After 1hr in normoxia at 37°C, cells were incubated under both normoxia and hypoxia as described above for a further 5 days prior to luciferase assay. For HIF-1α over-expression, PBMCs were transfected with 1ug of 240bp (-56 to +184) versican luciferase reporter construct, Phosphoglycerate kinase-1 luciferase reporter construct (PGK; constructed as described by Ameri et al., 2002) or empty pGL4.10 [luc2] plasmid (Promega). Cells were co-transfected with 300ng of HIF-1α over-expressing plasmid (pCDNA3.1/HIF-1α; a kind gift from Professor Chris Pugh, University of Oxford)) or the negative control plasmid (pCDNA 3.1 CAT) for 24 h in normoxia prior to luciferase assay. After incubation in either normoxia or hypoxia, medium was removed and cells were washed twice with 1xPBS. Cells were then lysed, and luciferase activities were quantified using luciferase assay reagents (Promega) according to the manufacturer’s protocol. Protein concentrations were measured using a Bradford assay (Thermo Scientific) and luciferase values were normalized to the obtained protein concentrations.

### Intracellular FACS analysis of versican protein expression

Based on previous work on quantitation of versican protein expression using FACS [44], HMDMs were incubated in low attachment plates (Costar) for 4 days under the appropriate oxygen tension, then incubated under the same oxygen tension for a further 24h in the presence of 2μg/ml Brefeldin A (B6542, Sigma) to block protein secretion prior to quantification of protein by FACS. Therefore the versican detected will represent newly synthesized protein, but possible contribution of some versican taken up from the cell supernatant can not be ruled out. Cells were fixed using 1% paraformaldehyde and permeabilised with 0.1% saponin (Sigma) in PBS, washed twice with PBS/2% FCS and incubated with monoclonal anti- human versican antibody (MAB3054, R&D Systems) or rat IgG_1_ isotype control (MAB005, R&D Systems) at 1:50 dilution on ice for 45 min. Cells were then washed and incubated with 1:40 diluted FITC conjugated anti-rat IgG (F1763, Sigma) on ice for 45 min. After washing, cells were acquired on a FACSCalibur flow cytometer and analyzed with CellQuest software (Becton Dickinson).

### Western Blots

Cells were detached from culture plates by scraping, pelleted at 400 *g* for 1 min at 4°C, resuspended in 4 volumes of ice cold lysis buffer (50mM Tris pH8, 280mM NaCl, 0.5% NP-40, 0.2mM EDTA, 2mM EGTA, 1mM PMSF, 0.1mM Na_3_VO_4_, 1mM DTT, 1μg/ml Leupeptin, 2μg/ml Aprotinin, and 1μg/ml Pepstatin—all reagents from Sigma), and incubated on ice for 5 min prior to storage at -80°C. Samples were assayed for protein using the D_C_ protein assay kit (BioRad, UK). Ten micrograms of protein was loaded onto Tris-glycine SDS page gels and electrophoresed and immunoblotted using specific antibodies at 1:250 dilution (HIF-1α: Transduction Laboratories Cat. No. 610959, Mouse monoclonal, Clone 54; Actin: Sigma Cat. No. A-2066, Rabbit polyclonal) as previously described [18].

### Database analysis

The versican promoter sequence was searched using Matlnspector (Genomatix, http://www.genomatix.de/online_help/help_matinspector/matinspector_help.html) and Transcription Element Search System (TESS) software (http://www.cbil.upenn.edu/cgi-bin/tess/tess?RQ=WELCOME).

### Statistics

Analyses were performed using GraphPad Prism, GraphPad Software Inc., San Diego, USA. Results were considered significant if *p* ≤ 0.05 (****, p ≤ 0.0001; ***, *p* ≤ 0.001; **, *p* ≤ 0.01; and *, *p* ≤ 0.05, ns = not significant).
